# Adaptation to a bacterial pathogen in *Drosophila melanogaster* is not aided by sexual selection

**DOI:** 10.1002/ece3.8543

**Published:** 2022-02-12

**Authors:** Sakshi Sharda, Tadeusz J. Kawecki, Brian Hollis

**Affiliations:** ^1^ Department of Ecology and Evolution University of Lausanne Lausanne Switzerland; ^2^ Department of Biological Sciences University of South Carolina Columbia South Carolina USA

**Keywords:** adaptation, condition dependence, good genes, Hamilton and Zuk hypothesis, pathogens, sexual selection

## Abstract

Theory predicts that sexual selection should aid adaptation to novel environments, but empirical support for this idea is limited. Pathogens are a major driver of host evolution and, unlike abiotic selection pressures, undergo epidemiological and co‐evolutionary cycles with the host involving adaptation and counteradaptation. Because of this, populations harbor ample genetic variation underlying immunity and the opportunity for sexual selection based on condition‐dependent “good genes” is expected to be large. In this study, we evolved populations of *Drosophila melanogaster* in a 2‐way factorial design manipulating sexual selection and pathogen presence, using a gram‐negative insect pathogen *Pseudomonas entomophila*, for 14 generations. We then examined how the presence of sexual selection and the pathogen, as well as any potential interaction, affected the evolution of pathogen resistance. We found increased resistance to *P*. *entomophila* in populations that evolved under pathogen pressure, driven primarily by increased female survival after infection despite selection for resistance acting only on males over the course of experimental evolution. This result suggests that the genetic basis of resistance is in part shared between the sexes. We did not find any evidence of sexual selection aiding adaptation to pathogen, however, a finding contrary to the predictions of “good genes” theory. Our results therefore provide no support for a role for sexual selection in the evolution of immunity in this experimental system.

## Graphical abstract

In this study, we set out to validate the “good genes” hypothesis related to pathogens, and we test the independent and interactive effects of sexual selection and pathogen presence on adaptation to pathogen in *D. melanogaster* populations by performing a full factorial experimental evolution experiment. We find that populations that evolved under pathogen pressure did evolve better survival toward the pathogen; however, this was driven by differences in female survival. Further, contrary to our expectations of good genes aiding adaptation in populations, we did not find any evidence of sexual selection aiding adaptation to pathogen, despite the pathogen leading to competitive differences between uninfected and infected males.

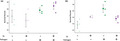

## 1 INTRODUCTION

Darwin posited that sexual selection plays an important role in improving non‐sexual fitness, writing that, “the strongest and most vigorous males, or those provided with the best weapons, have prevailed under nature, and have led to the improvement of the natural breed or species” (Darwin, 1871). The modern version of this idea proposes that sexually selected traits in males reflect “good genes” (Fisher, 1930; Houle & Kondrashov, 2002; Iwasa et al., 1991; Zahavi, 1975), explaining potentially costly female choice by indirect benefits received in the form of increased offspring fitness. Theory suggests expression of sexually selected traits should evolve to become dependent on overall condition—which would maintain signal fidelity—leading to accelerated rates of adaptation (Lorch et al., 2003) and more efficient purging of deleterious mutations (Whitlock & Agrawal, 2009).

In line with predictions of positive effects of sexual selection (Cally et al., 2019) on population performance, sexual selection has been found to diminish the likelihood of population extinction (Jarzebowska & Radwan, 2010; Lumley et al., 2015). Experimental work in different insect taxa including *Drosophila* has also shown that the presence of sexual selection accelerates the purging of deleterious alleles in experimental populations (Grieshop et al., 2016; Hollis et al., 2009; Radwan, 2004). In several experiments, sexual selection facilitated adaptation to novel environmental challenges, including the evolution of desiccation resistance in *D*. *melanogaster* (Gibson Vega et al., 2020), pesticide resistance in *Tribolium castaneum* (Jacomb et al., 2016), and adaptation to a novel diet in *Callosobruchus maculatus* (Fricke & Arnqvist, 2007). However, an arguably larger body of experimental work has found no role for sexual selection in improving non‐sexual fitness. Multiple experimental evolution studies failed to find population‐level net benefits of sexual selection when examining larval competitive ability, net reproductive rate, or female fecundity (Holland & Rice, 1999; Long et al., 2009; Promislow et al., 1998 respectively). Moreover, a large body of work has also failed to demonstrate a role of sexual selection in adaptation in novel environments (e.g., to higher temperatures (Holland, 2002) or a novel diet (Rundle et al., 2006)). There is also no evidence that overall mutation load from the genome is reduced under heightened sexual selection (Arbuthnott & Rundle, 2012; Hollis & Houle, 2011) (although in environments that are spatially complex, this is not true and the predicted beneficial effects of sexual selection on mutation load are seen (Singh et al., 2017)). Thus, taken together, the literature is equivocal about role of sexual selection in non‐sexual fitness. This leaves an open question about whether the “good genes” mechanism plays a role in adaptation in general or even in specific scenarios, such as during adaptation to pathogens or parasites, where this role has been predicted to be most evident but remains largely untested.

One potential explanation for these mixed results is that the non‐sexual fitness of populations is normally elevated by competition for mates—that is, sexual selection in the broad sense does have adaptive value—but these benefits are counterbalanced by the negative effects of sexual conflict and therefore invisible in many experimental designs. Sexual conflict arises because of an evolutionary conflict of interests between the sexes (Hosken et al., 2019; Parker, 1979) which can manifest in two ways. The first, interlocus sexual conflict, is characterized by selection favoring traits that increase male competitive success even when these traits are accompanied by harm to females. Interlocus sexual conflict can lead to the evolution of female resistance and sexually antagonistic coevolution (Chapman et al., 2003; Holland & Rice, 1999a; Rice et al., 2006), reducing mean population fitness (Bonduriansky & Chenoweth, 2009; Long et al., 2009, 2012). In *Drosophila*, interlocus sexual conflict acts through antagonistic effects on female fecundity and survival (Chapman, 2006; Rice, 1996), especially on the most fecund females (Long et al., 2009). Intralocus conflict, on the contrary, involves sexually antagonistic pleiotropic effects of polymorphisms at the same locus in males and females (Bonduriansky & Chenoweth, 2009; Innocenti & Morrow, 2010; Van Doorn, 2009) that constrain males and females from reaching sex‐specific optima (Chippindale, 2001; Hollis et al., 2014, 2019). Either form of sexual conflict leads to a burden on populations that might overwhelm any positive effects of sexual selection for mean population fitness (Bonduriansky & Chenoweth, 2009; Long et al., 2009, 2012).

Male–male competition and female choice have been proposed to be particularly consequential for evolution of pathogen resistance (Folstad & Karter, 1992; Hamilton & Zuk, 1982; Roberts et al., 2004). Pathogens are a major evolutionary driver of the life histories of organisms (Price, 1980; Schmid‐Hempel, 2005) due to their prevalence, diversity, and because they adapt to the host and represent a moving target for the immune system. According to the Hamilton–Zuk hypothesis (1982), sexual ornaments indicate immunity toward prevalent pathogens or parasites (Hamilton & Zuk, 1982; Martin, 1990). A number of studies in birds have indeed demonstrated phenotypic correlations between male parasite or pathogen load and the quality of sexual ornaments (Balenger & Zuk, 2014; Hamilton & Zuk, 1982; Martin, 1990) or female preference toward the males (Blount et al., 2003; Hund et al., 2020). Yet, whether this phenotypic correlation should be positive or negative is not unequivocally predicted by mathematical models; either may be predicted depending on details of the model assumptions (Getty, 2002). These phenotypic correlations between sexual ornaments and parasite/pathogen resistance do not necessarily predict whether sexually attractive fathers will sire resistant offspring; rather, this key element of the "good genes" hypothesis is mediated by additive genetic correlations (Hamilton & Zuk, 1982). One way to test for this genetic correlation would be to track the evolution of resistance under controlled laboratory conditions (Kawecki et al., 2012) where both the strength of sexual selection and pathogen pressure are manipulated. If there is an additive genetic correlation between sexually successful fathers and pathogen‐resistant offspring, resistance should evolve more readily in populations where males also experience sexual selection.

Selection for improved immunity (including better physiological responses to immune challenges) in experimental populations has generally resulted in a robust and rapid response (Armitage & Siva‐Jothy, 2005; Ferro et al., 2019; Joop et al., 2014; Martins et al., 2013). Two studies that explored the effect of sexual selection on immunity by experimentally evolving populations with and without sexual selection have found that males and females diverge in their investment in innate immunity (measured as phenyloxidase activity; PO) (Bagchi et al., 2021; Hangartner et al., 2015). In both studies (one on the flour beetle *Tribolium castaneum* and the other on the seed beetle *Callosobruchus maculatus*; (Hangartner et al., 2015; Bagchi et al., 2021, respectively)), females from polygamous populations had higher levels of PO than females from monogamous populations, with no effect on males from either of the two experimental regimes. The higher levels of PO in females from sexually selected populations did not influence pathogen clearance in either study, although in one of the studies, higher PO activity was correlated with lower survival in females upon bacterial infection (Bagchi et al., 2021). These studies indicate how sexual selection and sexual conflict can drive sex‐specific differences in male and female immunity. This pattern is not without exceptions; a study on the yellow dung fly, *Scathophaga stercoraria*, did not report sex differences in PO levels in populations evolved with or without sexual selection (Hosken, 2001). Hosken (2001) also found that monogamous populations had higher PO levels than polygamous populations, although here also this difference did not translate into differences in bacterial clearance after infection (Hosken, 2001). The above studies manipulated the presence or absence of either a pathogen or sexual selection. In the work reported here, we manipulated both pathogen and sexual selection in order to test for effects of the presence of each, as well as any interaction, on the evolution of pathogen resistance.

We carried out a 2‐way factorial evolutionary experiment manipulating sexual selection and exposure to a pathogen. We let replicate populations of *D*. *melanogaster* evolve for 14 generations either under controlled monogamy or random polygamy (i.e., with or without sexual selection; Hollis and Houle (2011)), each generation exposing males to either an intestinal pathogen (a gram‐negative bacterium *Pseudomonas entomophila*) or a sham treatment. In our experimental design, we only exposed males to the pathogen and allowed the males to interact with females beginning one day after exposure to the pathogen (we verified that males had cleared the bacteria from their gut at this timepoint and thus did not infect females). With this design, we aimed to increase the opportunity for sexual selection to act via differential mating success of males differentially coping with infection. We aimed to address several interconnected questions.

First, and most simply, do *D*. *melanogaster* populations exposed to the pathogen as adults evolve resistance, measured as survival after infection, over a short timescale? Resistance to *P*. *entomophila* has been reported to evolve after only four generations of strong selection imposed by breeding from flies that survived a prior infection (Martins et al., 2013). Second, if only one sex—in our design, males—experiences the pathogen, would evolved resistance to *P*. *entomophila* be detectable in the other sex? If evolved resistance is evident in both sexes, this would indicate a shared genetic basis. Third, would sexual selection lead to the evolution of differences in pathogen resistance even in the absence of pathogen? This would be predicted if there were an additive genetic correlation between male sexual traits and resistance that were expressed irrespective of pathogen exposure (Joye & Kawecki, 2019). A result supporting this prediction has been reported in *Tribolium* (Hangartner et al., 2015) and *Callosobruchus* (Bagchi et al., 2021); however, the conclusion was based on quantifying an aspect of immune response rather than resistance to an actual pathogen. Fourth, does sexual selection accelerate the evolution of resistance in populations exposed to the pathogen, and does it do so to a greater degree than would be expected based on the sum of effects of sexual selection and pathogen exposure acting alone? This positive interaction between the effects of pathogen and sexual selection would be expected if heritable variation in pathogen resistance influenced infected males' sexual success.

The rationale of this study relied on the pathogen affecting the sexual success of males. Therefore, prior to the evolutionary experiment, we tested whether infection with *P*. *entomophila* affects competitive paternity share. Mortality in our laboratory population was much lower than is generally reported (Faria et al., 2015; Joye & Kawecki, 2019; Martins et al., 2013), but uninfected males had greater competitive paternity success than infected males. If genetic variation conferring resistance to *P*. *entomophila* has a similar positive effect on male competitive success after exposure to the pathogen, this scenario should provide an opportunity for female choice to amplify non‐sexual selection and accelerate adaptation to pathogen.

## 2 MATERIALS AND METHODS

### 2.1 Stock populations and experimental conditions

The experimental populations were established from a long‐term laboratory population called Ives (IV) that was initiated from about 200 wild *D*. *melanogaster* of each sex collected in Massachusetts in 1975 (Charlesworth & Charlesworth, 1985). This population has been maintained in the laboratory at high density, with a census size in thousands, for more than 30 years and is adapted to the laboratory environment (Houle & Rowe, 2003). In the sexual competition experiment, we also used a reference population homozygous for a recessive *ebony* mutation previously backcrossed into the IV stock. To estimate pathogen virulence during experimental evolution, at each generation we ran a control using a line homozygous for a recessive *relish* mutation. The *relish* mutation blocks the Imd pathway that plays an important role in defense against gram‐negative bacterial pathogens (Hedengren et al., 1999); *relish* mutants are therefore highly susceptible to *P*. *entomophila* (Vallet‐Gely et al., 2010).

All flies in the experiment were maintained on fly media composed of (for 1 L water): 6.2 g Agar powder (ACROS N. 400400050), 58.8 g Farigel wheat (Westhove N. FMZH1), 58.8 g yeast (Springaline BA10), 100 ml grape juice; 4.9 ml propionic acid (Sigma N. P1386), and 26.5 ml of methyl 4‐hydroxybenzoate (Nipagin M, VWR N. ALFAA14289.0) solution (400 g/L) in 95% ethanol. Populations were kept at 25°C with a 12:12 h (L/D) cycle.

### 2.2 Sexual success of infected versus uninfected males

To determine whether infection has any effect on male sexual success, we compared the competitive paternity success of infected and sham‐treated males (infection protocol described below). Because the infected and uninfected males came from the same population, we would not be able to distinguish paternity in direct competitions. We therefore competed each against males from a reference population homozygous for the *ebony* marker.

Each replicate consisted of five focal males (either infected (*N* = 38) or sham‐treated (*N* = 39)) and five *ebony* males competing for five *ebony* females. These flies were allowed to interact for 48 h before being discarded. The resulting offspring were scored upon emergence as adults. The recessive *ebony* mutation enabled us to distinguish offspring sired by the focal males (which would have wild‐type cuticles) and those sired by the reference males (which would have dark cuticles). The proportion of wild‐type offspring was then used as a measure of sexual success of the infected versus non‐infected focal males. Even though the fraction of wild‐type offspring may deviate from the actual fertilization success of focal males because of differences in egg‐to‐adult survival of wild‐type and ebony offspring, this would affect the estimates for the two types of males in the same way.

### 2.3 Experimental regimes and selection protocol

To study the interplay between sexual selection (SS) and pathogen presence (P), we used a factorial design that manipulated the presence or absence of SS (polygamous versus monogamous mating systems) and the presence or absence of our pathogen, *P. entomophila*, resulting in 4 experimental regimes (+SS +P, +SS −P, −SS +P, and −SS −P). Within each experimental regime, 3 replicate populations were established. To establish experimental populations, adults were obtained by amplifying flies from the IV base population stock, collecting virgin flies, and randomly assigning 80 males and 80 females to each of the 12 populations. At 5–6 days old, virgin males were orally infected with *P*. *entomophila* (protocol described in the following section) in +P treatments and sham‐infected in −P treatments. Males were mated with virgin females for 72 h after being exposed to infection for 24 h. Under the +SS experimental regimes, groups of 5 males and 5 age‐matched virgin females were placed in interaction vials. Under the −SS regimes, groups of 1 male and 1 age‐matched virgin female were placed in interaction vials. Flies were left in these interaction vials for 72 h, after which mated females from each population were pooled and re‐distributed in groups of 20 to new vials for egg laying. Females were allowed to lay eggs for 72 h, after which they were discarded from the vials while larvae developed. The density of mated females was therefore controlled in the egg‐laying vials, but we did not further control for egg density, which appeared qualitatively the same across regimes and populations throughout the course of experimental evolution. We collected virgins from all experimental populations on Days 12 and 13 (and occasionally on Day 14) from the start of egg laying. Although there was some adult emergence in the days before and after, these collections corresponded to the peak eclosion time and minimized the chance we inadvertently selected for faster or slower development time. On emergence, virgins were collected and housed in groups of 20 in single‐sex vials until they were 5–6 days old, at which point the experimental protocol was repeated. Populations were maintained under the experimental regimes for 14 generations at a population size of 160 individuals (80 males + 80 females).

### 2.4 Infections

The pathogen used in our experiments, *P*. *entomophila*, is a naturally occurring gram‐negative bacteria isolated from *D*. *melanogaster* in Guadeloupe (Liehl et al., 2006; Vodovar et al., 2005). It is acquired during feeding and at high doses kills about 60% of *D*. *melanogaster* adults within 72 h and almost 70% of larvae in 48 h (Liehl et al., 2006). It has been found to elicit both local and systemic immune responses involving a range of host responses including the secretion of specific anti‐microbial peptides, repair, and regeneration of epithelial cells in the gut as a result of damage caused by the pathogen (Liehl et al., 2006; Vodovar et al., 2005) and leads to large‐scale changes in gene expression in response to this pathogen (Chakrabarti et al., 2012). This system has been used to study the genetic basis of immunity (Bou Sleiman et al., 2015; Chakrabarti et al., 2012; Neyen et al., 2014) as well as in an evolutionary context in work looking at life‐history trade‐offs (Vijendravarma et al., 2015) and sexual selection (Joye & Kawecki, 2019).

We obtained an isolate of *P*. *entomophila* from Bruno Lemaitre (EPFL). Bacteria were plated from glycerol stocks 3 days prior to infection on standard LB‐agar plates supplemented with 1% milk and grown for two days at room temperature. On the day before the infection, a single colony was transferred to a 50‐ml Erlenmeyer pre‐culture flask with 12.5 ml LB and incubated for 8 h in a shaking incubator at 29°C and 180 rpm. The pre‐culture flask was then transferred to a 2‐L Erlenmeyer flask with 400 ml LB (or 1‐L Erlenmeyer with 200 ml LB), and the culture was incubated overnight in the same shaking incubator at 29°C and 180 rpm. On the next day, the bacterial culture was centrifuged at 2500 *g* at 4°C for 20 min. The pellet was re‐suspended and mixed with sucrose and water to obtain a final infection cocktail with an OD of 300. The sham treatment was performed with a 2.5% sucrose solution.

Oral infection was performed as previously described (Neyen et al., 2014). Flies were first starved for 4 h and then transferred to a vial with a filter paper layered over food and soaked with 150 µl of the bacterial cocktail. Males were left in these vials for 24–26 h after which they were transferred to interaction vials with females. Dead flies were counted at 2, 4, 20, and 24 h after pathogen exposure.

### 2.5 Bacterial load in infected males

To examine how fast *D*. *melanogaster* males clear the *P*. *entomophila* infection, we infected 1‐ to 2‐day‐old virgin males in groups of 20 individuals as described above. We then measured bacterial load of individual flies at 4, 8, and 24 h from the onset of the infection treatment, randomly choosing 2 infection vials to sample at each timepoint. We carefully removed survivors by light anesthesia and randomly selected 5 individuals.

Each individual fly was then placed in an Eppendorf tube containing small glass beads and 100 μl of 70% ethanol to surface sterilize the fly cuticle. The tube was inverted a few times to ensure proper mixing after which the 70% ethanol was removed and replaced by 100 μl of Luria broth (LB). We then placed the Eppendorf tubes on a Precellys bead ruptor for 30 s at 6000 rpm in order to homogenize the flies. The homogenate was then serially diluted to obtain concentrations of 1:10, 1:100, 1:1000, 1:10,000, and 1:100,000. We plated 3 μl of each of these dilutions in 5 replicates on a single LB plate containing 1% milk. The plates were left for 50 h at room temperature, and colonies from each dilution and replicate were counted. For each dilution and time point combination, we calculated an average count of the number of colonies for the 5 technical replicates (from each sample) followed by calculating the total colony‐forming units using the formula below:

Total Colony‐forming Units=Number of colonies for a given dilution×Dilution factor

### 2.6 Survival assays at generation 14

To assess adaptation to pathogen, two blocks of survival assays were done on males and females after 14 generations of experimental evolution. To avoid parental effects, we first reared individuals from all populations for one generation in a common garden. To establish the common garden, collected virgins were housed together in vials containing 20 males and 20 females. These individuals were allowed to mate for 72 h, after which males were discarded. Females (*N* = 120 per block) from these mating vials were then collected and housed together for 72 h in groups of 20 to lay eggs. After discarding the females, larvae were allowed to develop and emerging virgin males and females were collected and housed in single‐sex groups of 20 each. Virgins (age at infection: Batch 1 – 5–7 days, Batch 2 – 4–5 days) were exposed to *P*. *entomophila* in the same manner as described above in single‐sex groups of 20. After exposure to the pathogen for 24 h (OD_600nm_ of infections: Batch 1 – 280, Batch 2 – 300), individuals from each vial were transferred to fresh vials and per vial deaths were scored at 2, 4, 20, 24, 28 (the first time point after transfer to new vials), 44, 52, and 72 h after pathogen exposure. Alongside the infections, two vials were sham‐treated for each of the populations to serve as controls. In each block, we again used flies with a *relish* mutation to ensure that the pathogen was virulent (Vallet‐Gely et al., 2010).

### 2.7 Statistical analysis

We performed all statistical analyses in R v3.4.3 with the package afex (Singmann et al., 2015), a wrapper for lme4 (Bates et al., 2011). We fit generalized linear mixed models (glmer) with the binomial family (logit link) where the response was the phenotype of each emerging fly (wild type or ebony, binary) in competitive mating success assays or the survival status of each fly (alive or dead; survival 72 h post‐infection) in the survival assays after 14 generations of experimental evolution. For the latter, we fit one model that included all the data (both male and female survival) and included effects of sexual selection, pathogen presence, sex, and all interactions. We also fit simpler models on sex‐specific subsets of the data that excluded an effect of sex. In all models, we included experimental block, population, and vial (nested within population) as random effects.

## 3 RESULTS

**FIGURE 1:**
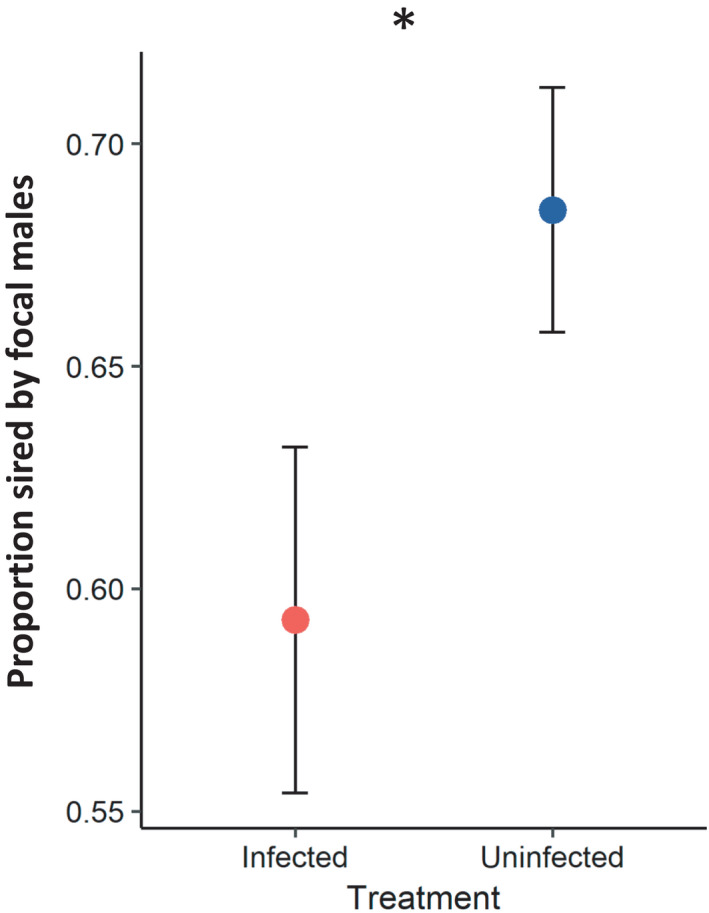
Competitive reproductive success of infected versus uninfected wild‐type males in an assay including both pre‐ and post‐copulatory effects. Paternity success is measured relative to a marked standard competitor

To assess the potential for sexual selection to act on pathogen resistance, we first compared the paternity success of infected and sham‐treated males in competition with males from a reference strain. We found that infected males had lower competitive mating success than uninfected males, as evidenced by a lower proportion of offspring sired by the focal males (treatment effect: $\chi_{df\;=1}^{2}$ = 4.45; *p* = .03; Figure 1, Table 1). Infected males sired on average 59.2% of progeny in competition with the competitive standard, while uninfected males sired on average 68.5% of progeny in competition with the competitive standard. This result indicated that infection harms male mating success and suggested that genetic variation contributing to infection resistance might be favored by sexual selection.

**TABLE 1:** Models for survival assays at 72 h post‐infection with *P*. *entomophila* after one generation of common garden rearing after Generation 14

Model	Intercept	*df*	*Χ^2^*	*p*
Generation 14				
Survival (full model)				
SS	−2.24	1	0.01	.91
Pathogen		1	8.89	***.002***
Sex		1	245.32	<2.2e^−16^
SS:Pathogen		1	1.33	.24
SS:Sex		1	0.14	.70
Pathogen:Sex		1	0.0074	.93
SS:Pathogen:Sex		1	5.97	***.014***
Male survival	−2.78			
Sexual selection		1	0.094	.75
Pathogen		1	3.40	.065
Sexual selection × Pathogen		1	4.71	***.029***
Female survival	−1.72			
Sexual selection		1	0.04	.82
Pathogen		1	4.92	***.026***
Sexual selection × Pathogen		1	0.93	.33

Note

*p* values  < .05 are formatted in Bold‐italics.

We also verified that the infected males had cleared the pathogen from their gut by the time they were placed with females. Although males harbored many live *P*. *entomophila* 4 h after the onset of the infection treatment, no live bacteria were detected at 8 or 24 h (Figure S1), in agreement with earlier results (Bou Sleiman et al., 2015). Thus, there was little opportunity for the males to transmit the infection to the females. Bacterial clearance from male guts does not preclude ongoing systemic and immune responses in males resulting from infection, however, making it plausible that males experience lasting effects of infection on sexual success.

We next evolved replicate populations with and without both sexual selection and pathogen for fourteen generations. Over the course of experimental evolution, *P*. *entomophila* virulence varied; the pathogen reliably killed a substantial fraction of the *relish* mutants (mean survival post‐infection 43% ± 10.7 (SE) in *relish* mutants; Figure S2). Survival at 24 h was lower in experimental populations exposed to the pathogen (+P), averaging 92.4%, than it was in populations not exposed to the pathogen (−P), in which survival was 99.9%.

**FIGURE 2:**
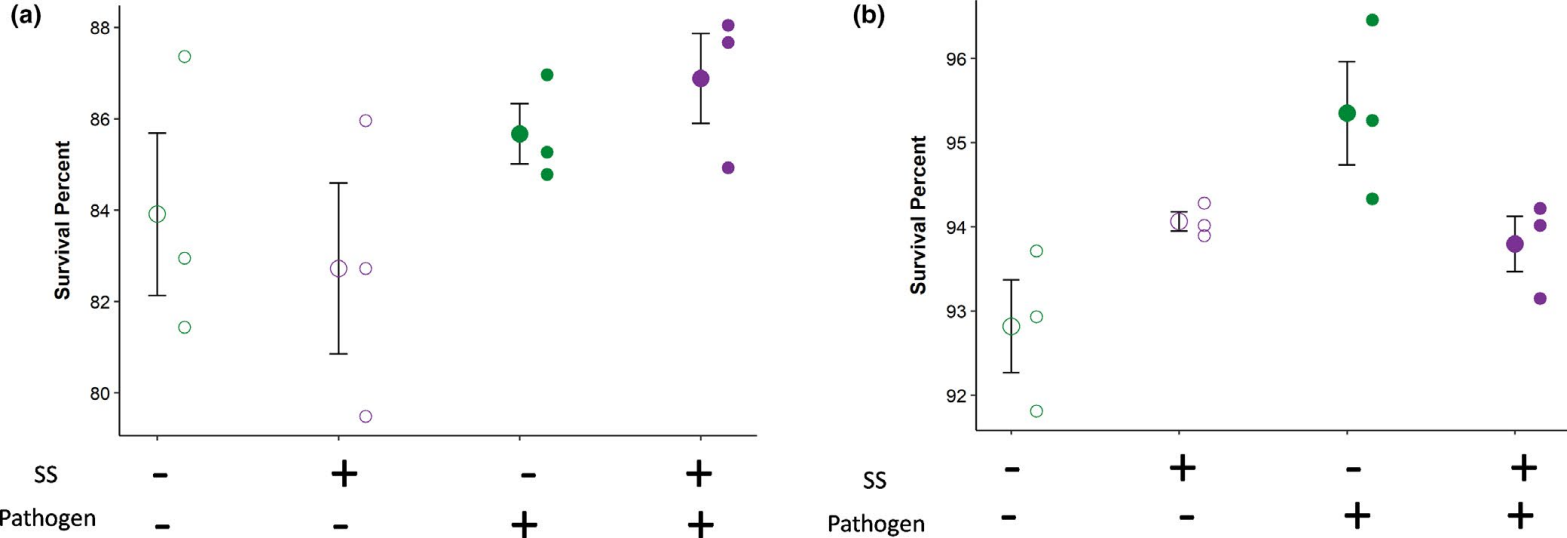
Survival at 72 h post‐infection with *P*. *entomophila*, for females (a) and males (b) pooled for both batches. Larger circles indicate the mean (± SE) of each evolutionary regime, while the smaller points represent the replicate populations within each regime

To compare resistance to *P*. *entomophila* in the populations subject to the different regimes, we measured their survival following infection after fourteen generations of experimental evolution and one generation of common garden rearing. In general, females survived less well after infection than males (Figure 2). Populations evolved under pathogen pressure (+P evolutionary regimes) showed better post‐infection survival than populations evolved without pathogen exposure (−P evolutionary regimes) (pathogen selection effect: $\chi_{df\;=1}^{2}$ = 8.89; *p* = .002; Figure 2, Table 1). A significant three‐way interaction between sexual selection, pathogen, and sex (SS * Pathogen * Sex, $\chi_{df=1}^{2}$ = 5.91; *p* = .01) indicates a difference between males and females in how the interaction between sexual selection and pathogen presence affects post‐infection survival, which we further explored in sex‐specific analyses.

In females, the sex‐specific analyses showed that post‐infection survival under +P regimes was better than that in the −P regimes (Figure 2a, pathogen selection effect: $\chi_{df=1}^{2}$ = 4.92; *p* = .026), but we detected no effect of sexual selection ($\chi_{df=1}^{2}$ = 0.04; *p* = .82) or any interaction between sexual selection and pathogen ($\chi_{df=1}^{2}$ = 0.93; *p* = .33). In males, there was neither a significant effect of sexual selection (Figure 2b, $\chi_{df=1}^{2}$ = 0.094; *p* = .75) nor pathogen ($\chi_{df=1}^{2}$ = 3.40; *p* = .06). However, there was a significant interaction between sexual selection treatment and pathogen presence ($\chi_{df=1}^{2}$ = 4.71; *p* = .029). For the good genes hypothesis to be true in our case, the +SS +P populations should have elevated survivorship compared with −SS +P regimes. However, in our study, we see the opposite effect, with the −SS +P regimes surviving significantly better than +SS +P (Figure 2b, Tukey's post hoc comparison *p* = .02). At the same time, there is no difference between +SS populations evolved with and without pathogen. This difference in the effect of sexual selection that depended on whether pathogen was present or not during the course of experimental evolution is what drives the significant interaction between sexual selection and pathogen.

## 4 DISCUSSION

In our study, we aimed to address the interplay of sexual selection and pathogen presence on the evolution of resistance to a pathogen, *P*. *entomophila*. We found a signature of pathogen resistance in populations evolved under pathogen pressure for fourteen generations when compared to populations evolved without it. Surprisingly, despite only infecting males over the course of experimental evolution, resistance to pathogen was more prominent in females. We did not find any evidence that sexual selection can promote the evolution of resistance to the pathogen, contrary to the predictions of theory (Hamilton & Zuk, 1982; Westneat & Birkhead, 1998). We expected that the presence of sexual selection and pathogen pressure would act synergistically, resulting in a greater response to selection and therefore improved survival post‐infection. We instead found an antagonistic interaction between the two in males, which could have possibly impeded the evolution of pathogen resistance.

Evolution of increased resistance of *D*. *melanogaster* to enteric infection and systemic infection has been seen in studies that have experimentally evolved fly populations with *P*. *entomophila* (Gupta et al., 2016; Martins et al., 2013). The study by Martins et al. (2013) imposed very strong selection on both sexes, with pathogen‐induced mortality up to 77% in the initial generations. In our experiment, pathogen selection was only applied on males and was associated with much lower mortality (5–25% depending on the generation). This lower virulence likely resulted from a difference in the bacterial genotype and/or the initial *Drosophila* gene pool; the IV population is generally robust and harbors high levels of genetic variation. It is likely that the overall strength of selection for resistance was therefore considerably lower in our experiment, but yet still sufficient to generate a detectable response. A stronger response to selection might have been obtained with a more virulent pathogen, or if both males and females had been infected each generation. Infecting females introduces a difficulty, however, in that reductions in female mating rate and fecundity make maintenance of experimental populations more challenging, and any reductions in female choosiness due to infection would be expected to diminish the importance of sexual selection. Lastly, it is also possible that effects of sexual selection and its interaction with the presence of pathogen, if present, would be detectable with a longer timescale as used in other studies (Fricke & Arnqvist, 2007; Rundle et al., 2006). However, the timescale used in our experiment was sufficient to detect both evolved survival differences in females from different regimes as well as an interaction of sexual selection with pathogen resistance in males that indicated a negative effect of sexual selection on adaptation to pathogen.

The fact that females from populations under pathogen pressure evolved higher resistance despite not experiencing direct selection supports a shared genetic basis for immunity between the sexes. Indeed, in line with this idea (Collet et al., 2016; Connallon & Hall, 2016), adaptation to desiccation resistance in experimentally evolved populations of *D*. *melanogaster* was observed both in males and females even when selection was imposed only on males (Gibson Vega et al., 2020). Adaptation in our experiment may also be more evident in female post‐infection survival simply because females show generally lower survival upon infection relative to males, which would make any evolved differences in survival easier to detect in females than males. Moreover, it is also plausible that alleles contributing to immunity that were favored in males under pathogen pressure had a larger effect size on resistance in females, making female resistance toward pathogen more detectable in this sex. We can exclude the possibility that selection did in fact act directly on females, for example, by sexual or social transmission of the pathogen from males to females, because the pathogen was cleared by males by the time they encountered females. However, clearance of pathogen post‐infection from male guts does not preclude an ongoing immune response resulting from infection. This ability of a male to tackle the pathogen and mount a systemic or local immune response could have been a target of sexual selection.

In our study, we do not see any evidence that sexual selection aids the evolution of resistance to pathogen. This, however, does not preclude the possibility that there might have been benefits conferred by sexual selection in +SS regimes. Previous studies have attributed the lack of adaptation to novel environments to the negative effects of sexual conflict (Holland & Rice, 1999b; Rundle et al., 2006). In a scenario where sexual conflict and sexual selection exert equal but opposing effects, both +SS −P and −SS +P regimes could show similar levels of adaptation. However, if sexual conflict negatively affected adaptation in our populations, we would have expected to find that populations exposed to the pathogen each generation but not experiencing sexual selection (−SS +P) would show a stronger signal of adaptation to pathogen than those exposed to pathogen and experiencing sexual selection (+SS +P). While our results on male survival after infection align with this idea, there is no signal of a cost to sexual selection in female survival after infection, leaving it difficult to attribute any importance to sexual conflict in our experiment.

In conclusion, our study found that populations of *D*. *melanogaster* evolved resistance to the insect pathogen *P*. *entomophila*, but this was either not facilitated (in females) or hindered (in males) by sexual selection. We expect that the low mortality in our study compared with previous work on this pathogen (Gupta et al., 2013, 2016; Joye & Kawecki, 2019; Martins et al., 2013), in which the majority of infected individuals die, provided a level of biological realism. The pathogen was still virulent enough to induce downstream effects on male sexual success, suggesting that genetic variation conferring resistance to pathogen would provide a large target for sexual selection. In addition, because most males survived infection during the course of experimental evolution, this provided an opportunity for sexual selection to reinforce non‐sexual selection by magnifying more subtle differences in pathogen resistance (e.g., differences in male condition or vigor that might emerge after weathering the infection). Despite a scenario that seems favorable for the detection of putative benefits of sexual selection—a relatively mild pathogen that might persist in natural host populations, that still yet influences mating success, in a host that harbors genetic variation for resistance—we found no such benefits.

## CONFLICT OF INTEREST

We declare that we have no competing interests.

## AUTHOR CONTRIBUTIONS

**Sakshi Sharda:** Conceptualization (equal); data curation (equal); formal analysis (equal); investigation (lead); methodology (equal); writing – original draft (equal); writing – review & editing (equal). **Tadeusz J. Kawecki:** Funding acquisition (equal); resources (equal); supervision (equal); writing – original draft (equal); writing – review & editing (equal). **Brian Hollis:** Conceptualization (equal); data curation (equal); formal analysis (equal); funding acquisition (equal); methodology (equal); resources (equal); supervision (equal); writing – original draft (equal); writing – review & editing (equal).

## Supporting information

Fig S1‐S2

## Data Availability

Data are available on dryad digital repository on this link—https://doi.org/10.5061/dryad.6djh9w12w.

## References

[ece38543-bib-0001] Arbuthnott, D. , & Rundle, H. D. (2012). Sexual selection is ineffectual or inhibits the purging of deleterious mutations in *Drosophila melanogaster* . Evolution (N.Y.), 66, 2127–2137. 10.1111/j.1558-5646.2012.01584.x 22759290

[ece38543-bib-0002] Armitage, S. A. O. , & Siva‐Jothy, M. T. (2005). Immune function responds to selection for cuticular colour in *Tenebrio molitor* . Heredity (Edinb), 94, 650–656. 10.1038/sj.hdy.6800675 15815710

[ece38543-bib-0003] Bagchi, B. , Corbel, Q. , Khan, I. , Payne, E. , Banerji, D. , Liljestrand‐Rönn, J. , Martinossi‐Allibert, I. , Baur, J. , Sayadi, A. , Immonen, E. , Arnqvist, G. , Söderhäll, I. , & Berger, D. (2021). Sexual conflict drives micro‐ and macroevolution of sexual dimorphism in immunity. BMC Biology, 19, 114. 10.1186/s12915-021-01049-6 34078377 PMC8170964

[ece38543-bib-0004] Balenger, S. L. , & Zuk, M. (2014). Testing the Hamilton‐Zuk hypothesis: Past, present, and future. Integrative and Comparative Biology, 54, 601–613. 24876194 10.1093/icb/icu059

[ece38543-bib-0005] Bates, D. , Maechler, M. , & Bolker, B. (2011). lme4: Linear mixed‐effects models using S4 classestle.

[ece38543-bib-0006] Blount, J. D. , Metcalfe, N. B. , Birkhead, T. R. , & Surai, P. F. (2003). Carotenoid modulation of immune function and sexual attractiveness in zebra finches. Science (80‐), 300, 125–127. 10.1126/science.1082142 12677066

[ece38543-bib-0007] Bonduriansky, R. , & Chenoweth, S. F. (2009). Intralocus sexual conflict. Trends in Ecology & Evolution, 24(5), 280–288. 19307043 10.1016/j.tree.2008.12.005

[ece38543-bib-0008] Bou Sleiman, M. S. , Osman, D. , Massouras, A. , Hoffmann, A. A. , Lemaitre, B. , & Deplancke, B. (2015). Genetic, molecular and physiological basis of variation in *Drosophila* gut immunocompetence. Nature Communications, 6, 7829. 10.1038/ncomms8829 PMC4525169 26213329

[ece38543-bib-0009] Cally, J. G. , Stuart‐Fox, D. , & Holman, L. (2019). Meta‐analytic evidence that sexual selection improves population fitness. Nature Communications, 10, 1–10. 10.1038/s41467-019-10074-7 PMC6494874 31043615

[ece38543-bib-0010] Chakrabarti, S. , Liehl, P. , Buchon, N. , & Lemaitre, B. (2012). Infection‐induced host translational blockage inhibits immune responses and epithelial renewal in the *Drosophila* gut. Cell Host & Microbe, 12, 60–70. 10.1016/j.chom.2012.06.001 22817988

[ece38543-bib-0011] Chapman, T. (2006). Evolutionary conflicts of interest between males and females. Current Biology, 16(17), R744–R754. 16950101 10.1016/j.cub.2006.08.020

[ece38543-bib-0012] Chapman, T. , Arnqvist, G. , Bangham, J. , & Rowe, L. (2003). Sexual conflict. Trends in Ecology & Evolution, 18, 41–47. 10.1016/S0169-5347(02)00004-6

[ece38543-bib-0013] Charlesworth, B. , & Charlesworth, D. (1985). Genetic variation in recombination in *Drosophila*. I. Responses to selection and preliminary genetic analysis. Heredity (Edinb), 54, 71–83. 10.1038/hdy.1985.10

[ece38543-bib-0014] Chippindale, A. K. , Gibson, J. R. , & Rice, W. R. (2001). Negative genetic correlation for adult fitness between sexes reveals ontogenetic conflict in *Drosophila* . Proceedings of the National Academy of Sciences, 98, 1671–1675. 10.1073/pnas.98.4.1671 PMC29315 11172009

[ece38543-bib-0015] Collet, J. M. , Fuentes, S. , Hesketh, J. , Hill, M. S. , Innocenti, P. , Morrow, E. H. , Fowler, K. , & Reuter, M. (2016). Rapid evolution of the intersexual genetic correlation for fitness in *Drosophila melanogaster* . Evolution (N.Y.), 70, 781–795. 10.1111/evo.12892 PMC5069644 27077679

[ece38543-bib-0016] Connallon, T. , & Hall, M. D. (2016). Genetic correlations and sex‐specific adaptation in changing environments. Evolution (N.Y.), 70, 2186–2198. 10.1111/evo.13025 27477129

[ece38543-bib-0017] Darwin, C. , Murray, J. , & William Clowes and Sons (1871). The descent of man: And selection in relation to sex. John Murray.

[ece38543-bib-0018] Faria, V. G. , Martins, N. E. , Paulo, T. , Teixeira, L. , Sucena, É. , & Magalhães, S. (2015). Evolution of *Drosophila* resistance against different pathogens and infection routes entails no detectable maintenance costs. Evolution (N.Y.), 69, 2799–2809. 10.1111/evo.12782 26496003

[ece38543-bib-0019] Ferro, K. , Peuß, R. , Yang, W. , Rosenstiel, P. , Schulenburg, H. , & Kurtz, J. (2019). Experimental evolution of immunological specificity. Proceedings of the National Academy of Sciences of the United States of America, 116, 20598–20604. 10.1073/pnas.1904828116 31548373 PMC6789748

[ece38543-bib-0020] Fisher, R. A. (1930). The genetical theory of natural selection (2nd ed.), Dover, 1958.

[ece38543-bib-0021] Folstad, I. , & Karter, A. J. (1992). Parasites, bright males, and the immunocompetence handicap. American Naturalist, 139, 603–622.

[ece38543-bib-0022] Fricke, C. , & Arnqvist, G. (2007). Rapid adaptation to a novel host in a seed beetle (*Callosobruchus maculatus*): The role of sexual selection. Evolution (N.Y.), 61, 440–454. 10.1111/j.1558-5646.2007.00038.x 17348953

[ece38543-bib-0023] Getty, T. (2002). Signaling health versus parasites. American Naturalist, 159, 363–371. 10.1086/338992 18707421

[ece38543-bib-0024] Gibson Vega, A. , Kennington, W. J. , Tomkins, J. L. , & Dugand, R. J. (2020). Experimental evidence for accelerated adaptation to desiccation through sexual selection on males. Journal of Evolutionary Biology, 33, 1060–1067. 10.1111/jeb.13634 32315476

[ece38543-bib-0025] Grieshop, K. , Stångberg, J. , Martinossi‐Allibert, I. , Arnqvist, G. , & Berger, D. (2016). Strong sexual selection in males against a mutation load that reduces offspring production in seed beetles. Journal of Evolutionary Biology, 29, 1201–1210. 10.1111/jeb.12862 26991346

[ece38543-bib-0026] Gupta, V. , Ali, Z. S. , & Prasad, N. G. (2013). Sexual activity increases resistance against *Pseudomonas entomophila* in male *Drosophila melanogaster* . BMC Evolutionary Biology, 13(1), 185. 24010544 10.1186/1471-2148-13-185 PMC3847581

[ece38543-bib-0027] Gupta, V. , Venkatesan, S. , Chatterjee, M. , Syed, Z. A. , Nivsarkar, V. , & Prasad, N. G. (2016). No apparent cost of evolved immune response in *Drosophila melanogaster* . Evolution (N.Y.), 70, 934–943. 10.1111/evo.12896 26932243

[ece38543-bib-0028] Hamilton, W. D. , & Zuk, M. (1982). Heritable true fitness and bright birds: A role for parasites? Science (80‐), 218, 384–387. 10.1126/science.7123238 7123238

[ece38543-bib-0029] Hangartner, S. , Michalczyk, Ł. , Gage, M. J. G. , & Martin, O. Y. (2015). Experimental removal of sexual selection leads to decreased investment in an immune component in female *Tribolium castaneum* . Infection, Genetics and Evolution, 33, 212–218. 10.1016/j.meegid.2015.05.005 25958137

[ece38543-bib-0030] Hedengren, M. , Åsling, B. , Dushay, M. S. , Ando, I. , Ekengren, S. , Wihlborg, M. , Hultmark, D. (1999). Relish, a central factor in the control of humoral but not cellular immunity in *Drosophila* . Molecular Cell, 4, 827–837. 10619029 10.1016/s1097-2765(00)80392-5

[ece38543-bib-0031] Holland, B. (2002). Sexual selection fails to promote adaptation to a new environment. Evolution (N.Y.), 56, 721–730. 10.1111/j.0014-3820.2002.tb01383.x 12038530

[ece38543-bib-0032] Holland, B. , & Rice, W. R. (1999a). Experimental removal of sexual selection reverses intersexual antagonistic coevolution and removes a reproductive load. Proceedings of the National Academy of Sciences of the United States of America, 96, 5083–5088. 10.1073/pnas.96.9.5083 10220422 PMC21820

[ece38543-bib-0033] Holland, B. , & Rice, W. R. (1999b). Experimental removal of sexual selection reverses intersexual antagonistic coevolution and removes a reproductive load. Proceedings of the National Academy of Sciences of the United States of America, 96, 5083–5088. 10.1073/pnas.96.9.5083 10220422 PMC21820

[ece38543-bib-0034] Hollis, B. , Fierst, J. L. , & Houle, D. (2009). Sexual selection accelerates the elimination of a deleterious mutant in *Drosophila melanogaster* . Evolution (N.Y.), 63, 324–333. 10.1111/j.1558-5646.2008.00551.x 19154371

[ece38543-bib-0035] Hollis, B. , & Houle, D. (2011). Populations with elevated mutation load do not benefit from the operation of sexual selection. Journal of Evolutionary Biology, 24, 1918–1926. 10.1111/j.1420-9101.2011.02323.x 21658188 PMC3156275

[ece38543-bib-0036] Hollis, B. , Houle, D. , Yan, Z. , Kawecki, T. J. , & Keller, L. (2014). Evolution under monogamy feminizes gene expression in *Drosophila melanogaster* . Nature Communications, 5, 3482. 10.1038/ncomms4482 24637641

[ece38543-bib-0037] Hollis, B. , Koppik, M. , Wensing, K. U. , Ruhmann, H. , Genzoni, E. , Erkosar, B. , Kawecki, T. J. , Fricke, C. , & Keller, L. (2019). Sexual conflict drives male manipulation of female postmating responses in *Drosophila melanogaster* . Proceedings of the National Academy of Sciences of the United States of America, 116, 8437–8444. 30962372 10.1073/pnas.1821386116 PMC6486729

[ece38543-bib-0038] Hosken, D. J. (2001). Sex and death: Microevolutionary trade‐offs between reproductive and immune investment in dung flies. Current Biology, 11(10), R379–R380. 11378399 10.1016/s0960-9822(01)00211-1

[ece38543-bib-0039] Hosken, D. J. , Archer, C. R. , & Mank, J. E. (2019). Sexual conflict. Current Biology, 29(11), R451–R455. 31163156 10.1016/j.cub.2019.03.052

[ece38543-bib-0040] Houle, D. , & Kondrashov, A. S. (2002). Coevolution of costly mate choice and condition‐dependent display of good genes. Proceedings of the Royal Society B‐Biological Sciences, 269, 97–104. 10.1098/rspb.2001.1823 PMC1690858 11788042

[ece38543-bib-0041] Houle, D. , & Rowe, L. (2003). Natural selection in a bottle. American Naturalist, 161, 50–67. 10.1086/345480 12650462

[ece38543-bib-0042] Hund, A. K. , Hubbard, J. K. , Albrecht, T. , Vortman, Y. , Munclinger, P. , Krausová, S. , Tomášek, O. , & Safran, R. J. (2020). Divergent sexual signals reflect costs of local parasites. Evolution (N.Y.), 74, 2404–2418. 10.1111/evo.13994 32385910

[ece38543-bib-0043] Innocenti, P. , & Morrow, E. H. (2010). The sexually antagonistic genes of *Drosophila melanogaster* . PLoS Biology, 8, e1000335. 10.1371/journal.pbio.1000335 20305719 PMC2838750

[ece38543-bib-0044] Iwasa, Y. , Pomiankowski, A. , & Nee, S. (1991). The evolution of costly mate preferences. II. The “handicap” principle. Evolution (N.Y.), 45, 1431–1442. 10.1111/j.1558-5646.1991.tb02646.x 28563835

[ece38543-bib-0045] Jacomb, F. , Marsh, J. , & Holman, L. (2016). Sexual selection expedites the evolution of pesticide resistance. Evolution (N.Y.), 70, 2746–2751. 10.1111/evo.13074 27677862

[ece38543-bib-0046] Jarzebowska, M. , & Radwan, J. (2010). Sexual selection counteracts extinction of small populations of the bulb mites. Evolution (N.Y.), 64, 1283–1289. 10.1111/j.1558-5646.2009.00905.x 19930452

[ece38543-bib-0047] Joop, G. , Roth, O. , Schmid‐Hempel, P. , & Kurtz, J. (2014). Experimental evolution of external immune defences in the red flour beetle. Journal of Evolutionary Biology, 27, 1562–1571. 10.1111/jeb.12406 24835532

[ece38543-bib-0048] Joye, P. , & Kawecki, T. J. (2019). Sexual selection favours good or bad genes for pathogen resistance depending on males' pathogen exposure. Proceedings of the Royal Society B‐Biological Sciences, 286, 20190226. 10.1098/rspb.2019.0226 PMC6532500 31064300

[ece38543-bib-0049] Kawecki, T. J. , Lenski, R. E. , Ebert, D. , Hollis, B. , Olivieri, I. , & Whitlock, M. C. (2012). Experimental evolution. Trends in Ecology & Evolution, 27(10), 547–560. 22819306 10.1016/j.tree.2012.06.001

[ece38543-bib-0050] Liehl, P. , Blight, M. , Vodovar, N. , Boccard, F. , & Lemaitre, B. (2006). Prevalence of local immune response against oral infection in a *Drosophila*/*Pseudomonas* infection model. PLoS Path, 2, 0551–0561. 10.1371/journal.ppat.0020056 PMC1475658 16789834

[ece38543-bib-0051] Long, T. A. F. , Agrawal, A. F. , & Rowe, L. (2012). The effect of sexual selection on offspring fitness depends on the nature of genetic variation. Current Biology, 22, 204–208. 10.1016/j.cub.2011.12.020 22226747

[ece38543-bib-0052] Long, T. A. F. , Pischedda, A. , Stewart, A. D. , & Rice, W. R. (2009). A cost of sexual attractiveness to high‐fitness females. PLoS Biology, 7, e1000254. 10.1371/journal.pbio.1000254 19997646 PMC2780925

[ece38543-bib-0053] Lorch, P. D. , Proulx, S. , Rowe, L. , & Day, T. (2003). Condition‐dependent sexual selection can accelerate adaptation. Evolutionary Ecology, 5(6), 867–881.

[ece38543-bib-0054] Lumley, A. J. , Michalczyk, Ł. , Kitson, J. J. N. , Spurgin, L. G. , Morrison, C. A. , Godwin, J. L. , Dickinson, M. E. , Martin, O. Y. , Emerson, B. C. , Chapman, T. , & Gage, M. J. G. (2015). Sexual selection protects against extinction. Nature, 522, 470–473. 10.1038/nature14419 25985178

[ece38543-bib-0055] Martin, C. (1990). Parasites and sexual selection: Current Hamilton and Zuk hypothesis. Behavioral Ecology and Sociobiology, 328, 319–328.

[ece38543-bib-0056] Martins, N. E. , Faria, V. G. , Teixeira, L. , Magalhães, S. , & Sucena, É. (2013). Host adaptation is contingent upon the infection route taken by pathogens. PLoS Path, 9, e1003601. 10.1371/journal.ppat.1003601 PMC3784483 24086131

[ece38543-bib-0057] Neyen, C. , Bretscher, A. J. , Binggeli, O. , & Lemaitre, B. (2014). Methods to study *Drosophila* immunity. Methods, 68, 116–128. 10.1016/j.ymeth.2014.02.023 24631888

[ece38543-bib-0058] Parker, G. A. (1979). Sexual selection and sexual conflict. In M. S. Blum , & N. S. Blum (Eds.), Sexual selection and reproductive competition in insects. Academic Press Inc.

[ece38543-bib-0059] Price, P. W. (1980). Evolutionary biology of parasites. Princeton University Press.

[ece38543-bib-0060] Promislow, D. E. L. , Smith, E. A. , & Pearse, L. (1998). Adult fitness consequences of sexual selection in *Drosophila melanogaster* . Proceedings of the National Academy of Sciences of the United States of America, 95, 10687–10692. 10.1073/pnas.95.18.10687 9724765 PMC27956

[ece38543-bib-0061] Radwan, J. (2004). Effectiveness of sexual selection in removing mutations induced with ionizing radiation. Ecology Letters, 7, 1149–1154. 10.1111/j.1461-0248.2004.00681.x

[ece38543-bib-0062] Rice, W. R. (1996). Sexually antagonistic male adaptation triggered by experimental arrest of female evolution. Nature, 381, 232–234. 10.1038/381232a0 8622764

[ece38543-bib-0063] Rice, W. R. , Stewart, A. D. , Morrow, E. H. , Linder, J. E. , Orteiza, N. , & Byrne, P. G. (2006). Assessing sexual conflict in the *Drosophila melanogaster* laboratory model system. Philosophical Transactions of the Royal Society B: Biological Sciences, 361, 287–299. 10.1098/rstb.2005.1787 PMC1569610 16612888

[ece38543-bib-0064] Roberts, M. L. , Buchanan, K. L. , & Evans, M. R. (2004). Testing the immunocompetence handicap hypothesis: A review of the evidence. Animal Behaviour, 68(2), 227–239.

[ece38543-bib-0065] Rundle, H. D. , Chenoweth, S. F. , & Blows, M. W. (2006). The roles of natural and sexual selection during adaptation to a novel environment. Evolution (N.Y.), 60, 2218–2225. 10.1111/j.0014-3820.2006.tb01859.x 17236415

[ece38543-bib-0066] Schmid‐Hempel, P. (2005). Evolutionary ecology of insect immune defenses. Annual Review of Entomology, 50, 529–551. 10.1146/annurev.ento.50.071803.130420 15471530

[ece38543-bib-0067] Singh, A. , Agrawal, A. F. , & Rundle, H. D. (2017). Environmental complexity and the purging of deleterious alleles. Evolution (N.Y.), 71, 2714–2720. 10.1111/evo.13334 28840604

[ece38543-bib-0068] Singmann, H. , Bolker, B. , & Westfall, B. (2015). afex: Analysis of factorial experiments. R package version 0.15‐2.

[ece38543-bib-0069] Vallet‐Gely, I. , Novikov, A. , Augusto, L. , Liehl, P. , Bolbach, G. , Péchy‐Tarr, M. , Cosson, P. , Keel, C. , Caroff, M. , & Lemaitre, B. (2010). Association of hemolytic activity of *Pseudomonas entomophila*, a versatile soil bacterium, with cyclic lipopeptide production. Applied and Environment Microbiology, 76, 910–921. 10.1128/AEM.02112-09 PMC2812987 20023108

[ece38543-bib-0070] Van Doorn, G. S. (2009). Intralocus sexual conflict. Annals of the New York Academy of Sciences, 1168, 52–71. 10.1111/j.1749-6632.2009.04573.x 19566703

[ece38543-bib-0071] Vijendravarma, R. K. , Narasimha, S. , Chakrabarti, S. , Babin, A. , Kolly, S. , Lemaitre, B. , & Kawecki, T. J. (2015). Gut physiology mediates a trade‐off between adaptation to malnutrition and susceptibility to food‐borne pathogens. Ecology Letters, 18, 1078–1086. 10.1111/ele.12490 26249109

[ece38543-bib-0072] Vodovar, N. , Vinals, M. , Liehl, P. , Basset, A. , Degrouard, J. , Spellman, P. , Boccard, F. , & Lemaitre, B. (2005). *Drosophila* host defense after oral infection by an entomopathogenic Pseudomonas species. Proceedings of the National Academy of Sciences of the United States of America, 102, 11414–11419. 10.1073/pnas.0502240102 16061818 PMC1183552

[ece38543-bib-0073] Westneat, D. F. , & Birkhead, T. R. (1998). Alternative hypotheses linking the immune system and mate choice for good genes. Proceedings of the Royal Society B‐Biological Sciences, 265, 1065–1073. 10.1098/rspb.1998.0400

[ece38543-bib-0074] Whitlock, M. C. , & Agrawal, A. F. (2009). Purging the genome with sexual selection: Reducing mutation load through selection on males. Evolution, 63(3), 569–582. 19154364 10.1111/j.1558-5646.2008.00558.x

[ece38543-bib-0075] Zahavi, A. (1975). Mate selection ‐ A selection for a handicap. Journal of Theoretical Biology, 53, 205–214. 10.1016/0022-5193(75)90111-3 1195756

