# Supplementary material for: Adaptation to a bacterial pathogen in Drosophila melanogaster is not aided by sexual selection
Source: Ecol Evol. 2022 Feb 12;12(2):e8543. doi: 10.1002/ece3.8543 (PMC8840902; doi:10.1002/ece3.8543)
Supplement: Supplementary file 1 — Fig S1‐S2 [file ECE3-12-e8543-s001.docx]

**Supplemental information for**

**Title:**

**Adaptation to a bacterial pathogen in *Drosophila melanogaster* is not aided by sexual selection**

Sakshi Sharda^1^, Tadeusz J. Kawecki^1^, Brian Hollis^2^

^1^ Department of Ecology and Evolution, University of Lausanne, CH-1016 Lausanne, Switzerland

^2^ Department of Biological Sciences, University of South Carolina, Columbia SC 29208, USA

***Supplemental Figures***


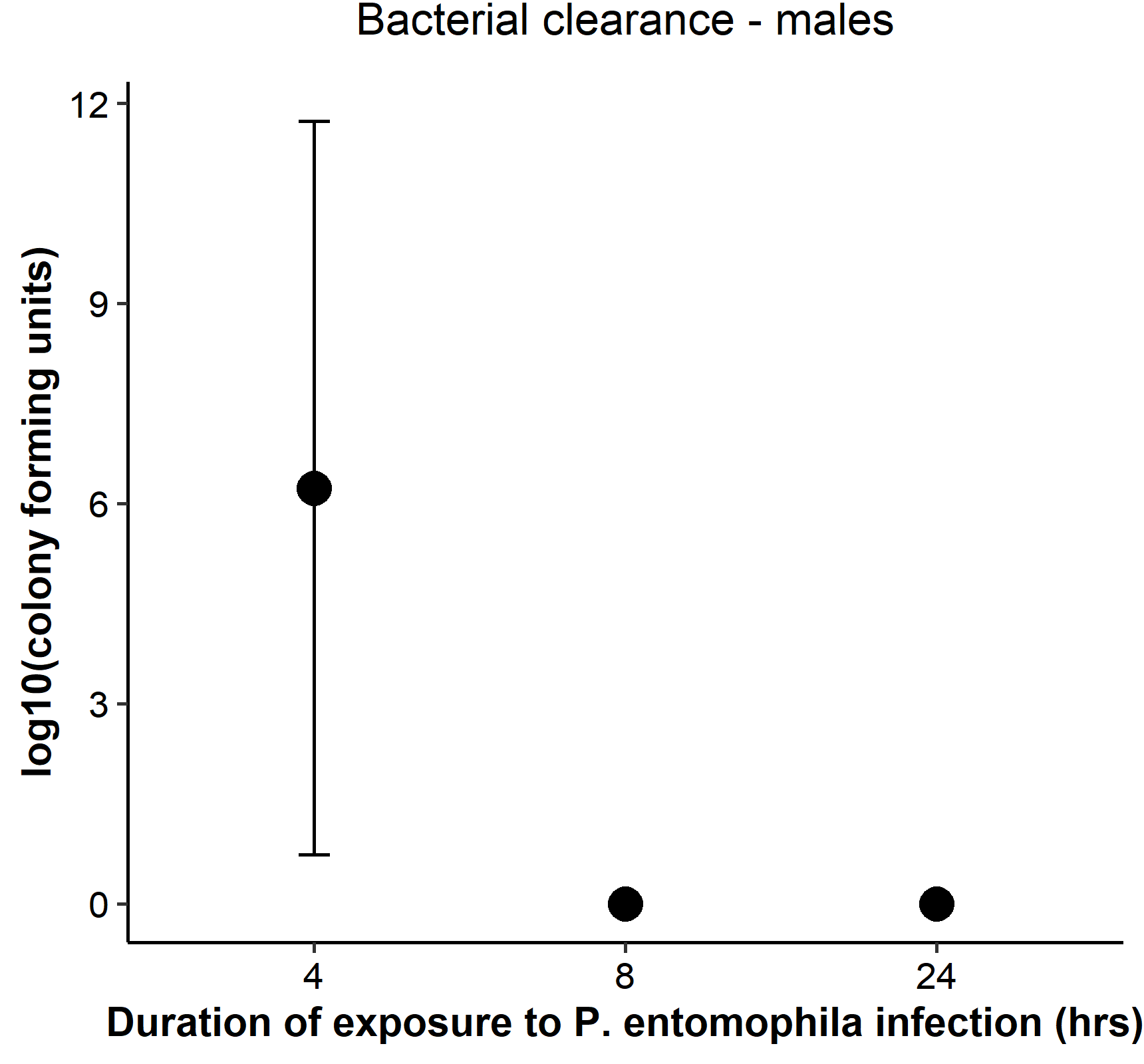


**Figure S1.** Internal bacterial load of virgin males exposed to *P. entomophila* following the infection protocol used in this study. The males harbor no detectable levels of live bacteria 8 hours from the onset of infection despite still being housed in the infection vials. Male survival in this experiment was similar to that seen during the course of the experimental evolution (survival at 24 hours: 94.5%).


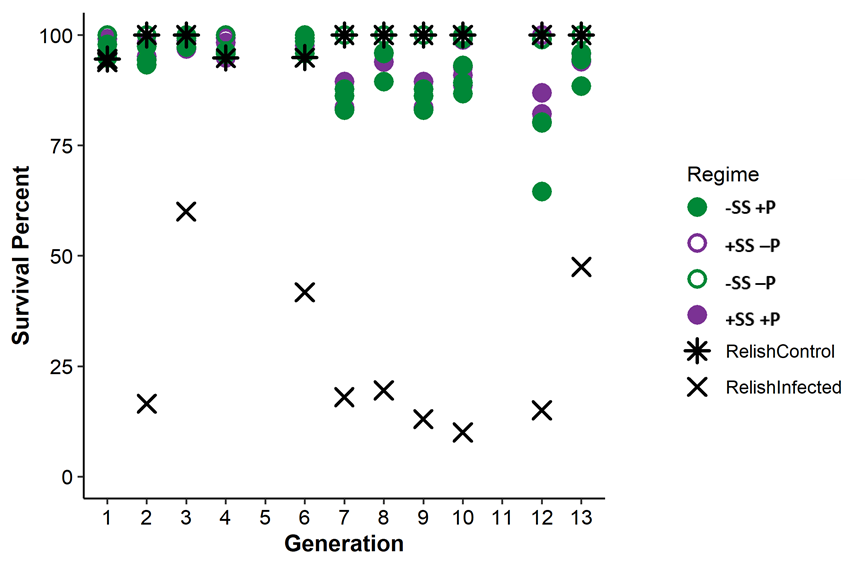


**Figure S2.** Survival of different experimental evolution lines under *P. entomophila* infection (+SS +P and –SS +P) and sham infection (+SS –P and –SS –P). At every generation, *relish* mutants were infected alongside the experimentally evolved lines to estimate the virulence of the pathogen. (Relish control refers to *relish* mutants exposed to sham infection to estimate background mortality in these lines.)
